# Plasma Protein and MicroRNA Biomarkers of Insulin Resistance: A Network-Based Integrative -Omics Analysis

**DOI:** 10.3389/fphys.2019.00379

**Published:** 2019-04-05

**Authors:** Hyungwon Choi, Hiromi W. L. Koh, Lihan Zhou, He Cheng, Tze Ping Loh, Ehsan Parvaresh Rizi, Sue Anne Toh, Gabriele V. Ronnett, Bevan E. Huang, Chin Meng Khoo

**Affiliations:** ^1^Department of Medicine, Yong Loo Lin School of Medicine, National University of Singapore, Singapore, Singapore; ^2^Saw Swee Hock School of Public Health, National University of Singapore, Singapore, Singapore; ^3^Institute of Molecular and Cell Biology, Agency for Science, Technology, and Research, Singapore, Singapore; ^4^MiRXES, Pte. Ltd., Singapore, Singapore; ^5^Department of Laboratory Medicine, National University Hospital, Singapore, Singapore; ^6^Janssen Research & Development US, World Without Disease Accelerator, Spring House, NJ, United States; ^7^Janssen Research & Development US, South San Francisco, CA, United States

**Keywords:** obesity, insulin resistance, proteomics, microRNAs, network analysis

## Abstract

Although insulin resistance (IR) is a key pathophysiologic condition underlying various metabolic disorders, impaired cellular glucose uptake is one of many manifestations of metabolic derangements in the human body. To study the systems-wide molecular changes associated with obesity-dependent IR, we integrated information on plasma proteins and microRNAs in eight obese insulin-resistant (OIR, HOMA-IR > 2.5) and nine lean insulin-sensitive (LIS, HOMA-IR < 1.0) normoglycemic males. Of 374 circulating miRNAs we profiled, 65 species increased and 73 species decreased in the OIR compared to the LIS subjects, suggesting that the overall balance of the miRNA secretome is shifted in the OIR subjects. We also observed that 40 plasma proteins increased and 4 plasma proteins decreased in the OIR subjects compared to the LIS subjects, and most proteins are involved in metabolic and endocytic functions. We used an integrative -omics analysis framework called iOmicsPASS to link differentially regulated miRNAs with their target genes on the TargetScan map and the human protein interactome. Combined with tissue of origin information, the integrative analysis allowed us to nominate obesity-dependent and obesity-independent protein markers, along with potential sites of post-transcriptional regulation by some of the miRNAs. We also observed the changes in each -omics platform that are not linked by the TargetScan map, suggesting that proteins and microRNAs provide orthogonal information for the progression of OIR. In summary, our integrative analysis provides a network of elevated plasma markers of OIR and a global shift of microRNA secretome composition in the blood plasma.

## Introduction

Insulin resistance (IR) is a state where a high concentration of insulin is needed to exert its normal biological functions, in particular in regulating glucose metabolism and maintaining the balance with lipid and protein metabolism. It is also described as an adaptive physiological response that accompanies many metabolic disorders such as T2D, hypertension, cardiovascular disease, dyslipidemia, and polycystic ovarian disease. IR is closely linked to obesity, but obesity only explains 22% of its variability (Abbasi et al., 2002). On the other hand, IR can be a manifestation of a systematic defensive response to nutrient excess to reduce cellular metabolic stress and formation of reactive oxygen species by limiting glucose entry into the cells (Hoehn et al., 2009). It is contemplated that the protective mechanism develops into a maladaptive response, leading to hyperglycemia together with many other dysregulated metabolic events.

A growing number of studies have already provided first snapshots of the molecular landscape of IR at the genome, transcriptome, proteome and metabolome level, dissecting the difference between obesity-dependent and obesity-independent pathways leading to IR. In a large-scale genome-wide association study of 188,577 individuals, Lotta et al. (2017) identified 53 genetic loci that are associated with a human IR phenotype, and hypothesized that these genetic loci might have influence on the capacity of peripheral adipose tissue to store fat in the pathogenesis of IR-related cardiometabolic disease. Frankie and Abbas showed that the transcriptome and proteome of the insulin signaling, gluconeogenic and inflammatory pathways in skeletal muscle and T-lymphocytes are significantly different between healthy subjects and patients with T2D (Stentz and Kitabchi, 2007).

At the plasma proteome level, various biomarkers secreted by immune cells, adipose tissues and liver have been associated with IR (Yki-Jarvinen, 2005; Kwon and Pessin, 2013; Lee and Lee, 2014). For example, low concentration of adiponectin (from adipose tissue) and IGFBP1 (from liver) in circulating blood has been consistently associated with IR and risk of glucose intolerance (Sandhu et al., 2002; Khoo et al., 2011). Higher inflammatory chemokines such as CRP, IL6, and TNF-α might affect skeletal muscle insulin signaling through endocrine and paracrine effects (Wei et al., 2008). Finally, several investigators have demonstrated the relationship between IR and the metabolome in the blood plasma, in particular circulating branched-chain amino acids, acylcarnitines and sphingolipids (Summers, 2010; Newgard, 2017).

The abundance of proteins associated with IR and obesity is also known to be tightly regulated at the post-transcriptional level by microRNAs (O’Hara et al., 2009). MicroRNAs (miRNAs) are small non-coding RNAs that regulate their target mRNAs in a sequence specific manner through mRNA degradation or translational silencing (Cannell et al., 2008). The relationships between miRNAs and the target genes constitute a complex network, as one miRNA can target multiple mRNAs, and multiple miRNAs can target the same mRNA, and their translational control occurs in a combinatorial manner (Cursons et al., 2018). The functional impact of miRNA-based gene expression regulation encompasses diverse biological pathways. Thus, the changes in global miRNA expression or the microRNAome are expected to play major roles in IR development and progression through adapted protein synthesis and mRNA turnover across the genome.

Circulating miRNAs also play a critical role in intercellular communication and signal transduction. They can be secreted from cells via RNA-binding proteins or exosomes and remain stable in the circulation, migrating into the recipient cells and controlling protein expression therein (Bayraktar et al., 2017). Relevant to metabolic disease, miRNAs have been shown to modulate key metabolic functions such as cholesterol and lipid metabolism (miR-33a, miR-122), insulin and glucose homeostasis (miR-375, miR-103, miR-107) and hepatic lipid metabolism (miR-34a) (Rottiers and Naar, 2012).

Despite the growing knowledge of the secretome changes in obesity and IR, few have profiled circulating proteins and miRNAs simultaneously from the same biospecimen and probed the regulatory role of miRNAs with the associated proteome. To address this gap, we profiled the circulatory proteins and miRNAs in OIR subjects and compared those to LIS subjects. We deliberately recruited obese IR subjects, as not all obese subjects are insulin resistant. We aimed to describe the landscape of differential proteins and miRNAs and provide an integrated map of the regulatory relationships between miRNAs and their target gene’s proteins with tissue of origin information for the latter.

## Materials and Methods

### Study Participants

The study methodology has been previously described in detail (Rizi et al., 2016). Briefly, we recruited nine obese (BMI ≥ 27.5 kg/m^2^) insulin-resistant (OIR) and nine lean (BMI ≥ 18.5 and ≤ 23 kg/m^2^) LIS Chinese males aged 21–40 years. All patients had fasting plasma glucose < 5.6 mmol/L and HbA1c < 6.0%. Subjects with HOMA-IR (HOMA-IR) ≥ 2.5 were considered as insulin resistant, and those with HOMA-IR < 1.2 as insulin sensitive. We excluded subjects with known first-degree family history of diabetes mellitus, prior history of pre-diabetes or diabetes mellitus, current thyroid disorders, history of malignancy, hospitalization or surgery within the past 6 months before the study, use of lipid-lowering, daily alcohol consumption exceeding 3 units, high level of physical activity (>5 h per week) or change in body weight ≥ 5% over the past 3 months. All subjects provided written consent before participation in the study. This study was carried out in accordance with the recommendations of ‘name of guidelines, name of committee’ with written informed consent from all subjects. All subjects gave written informed consent in accordance with the Declaration of Helsinki. The protocol was approved by the National Healthcare Group Domain Specific Review Board (Singapore).

### Clinical Measurements

We collected demographic data, medical and drug history. Height and weight were measured using a wall-mounted stadiometer and a digital scale, respectively. BMI was computed using the subject’s weight (in kg) divided by the square of his height (in m). HOMA-IR was calculated using the following formula: fasting insulin (mU/l) × fasting glucose (mmol/l)/22.5. Plasma glucose and insulin were measured using an enzymatic method (AU5800, Beckman Coulter, Inc., Brea, CA, United States) and a chemiluminescence immunoassay (ADVIA Centaur, Siemens Healthcare Diagnostics, Hamburg, Germany), respectively. The HbA1c was measured using the laboratory-based HPLC analyzer with CVs less than 2%.

### LC–MS Proteomics

Plasma proteomics experiments were performed by the Caprion Biosciences, Inc. All plasma samples went through standard sample preparation protocol including depletion of highly abundant proteins and trypsin digestion, followed by strong cation-exchange chromatography fractionation. Each fraction of a sample was analyzed by Q Exactive^TM^ mass spectrometer (Thermo Fisher) with separation through NanoAcquity UPLC-based liquid chromatography. For protein identification, the tandem mass spectra were searched against UniProt human protein database UP000005649 using Mascot software (Matrix Science, version 2.5.1) and peptides and associated protein IDs were identified at 6.4% false discovery rate. After cross-sample alignment by chromatographic retention time, peptides with 25% or higher frequency of missing data were removed, and the missing peptide intensities were imputed using the *K*-nearest neighbors method. The processed peptide intensity data was normalized so that the median intensity is the same across all the samples. Finally, protein intensity was derived by the sum of peptide-level intensities. See Supplementary Table S3.

### MicroRNA Profiling

374 miRNAs were profiled using the multiplexed RT-qPCR platform called MiRXES (MIRXES, Pte. Ltd., Singapore). The experimental protocol is as follows.

Total RNA was isolated from 200 uL plasma with Qiagen miRNeasy serum/plasma kit according to manufacturer’s protocol. Three spike-ins synthetic short RNAs with distinct sequence from endogenous human miRNAs were added in Qiazol as controls to normalize workflow variations. MS2 was also added into Qiazol as a carrier to prevent RNA loss during isolation step. Each 200 uL of plasma was mixed thoroughly with 1 ml of Qiazol, and then added with 200 μl of chloroform. Phase separation was performed at 4°C at 18000 *g* for 15 min, and the resulting 600 μl of aqueous phase was transferred to Qiacube for automated binding and washing. The resulting RNA was then eluted with 30 μl of nuclease free water. Another distinct set of three synthetic short RNAs that are not endogenous were added at this stage to further control for RT-qPCR efficiency.

Isolated plasma RNA was reverse-transcribed using the Xtensa miRNA reverse transcription (RT) kit (MiRXES) and modified stem-loop RT primer pools (MiRXES). The RT reaction was carried out at 42°C for 30 min, followed by 90°C for 5 min. For each RT reaction, a standard consisted of a serial of six 10-fold dilutions of synthetic miRNA and two no-template controls (NTCs) were reverse transcribed at the same time for all the samples. The standard was diluted in similar matrix as samples. cDNA was then pre-amplified before qPCR reaction to increase the concentration of templates for higher sensitivity and better dilution repeatability. All samples were pre-amplified for 17 cycles together with reverse transcribed standards. Subsequently, pre-amplified cDNA were diluted 100-fold before qPCR.

The qPCR reaction for each sample was performed in duplicates with the following protocol: 95°C for 10 min, followed by 40 cycles of 95°C for 10 s and 60°C for 30 s (optical reading). Raw Ct values were calculated using the ViiA 7 RUO software with automatic baseline setting and a threshold of 0.5 Ct values were averaged and compared against standard for the interpolation of copy number. Any miRNA with Ct number later than that of NTC was deemed undetectable. The data were further normalized so that the median and the standard deviation of log2 copy numbers of microRNAs were the same across all samples.

### Statistical Analysis

The statistical analyses of the anthropometric and biochemistry outcomes were performed using SPSS version 22.0 for Windows (SPSS, Inc., Chicago, IL, United States) using the Student’s *t*-test, with and without adjustment for age. Each -omics data set was first analyzed by principal component analysis (PCA) using prcomp command in R^1^. Differential expression analysis of both types of molecular data was performed using two-sample *t*-test (t.test command from stats library) and multiple testing correction by *q*-values (Bioconductor package *q*-value) (Diz et al., 2011). Volcano plots and heatmaps were produced using custom R scripts. Proteins and miRNAs with *q*-values below 0.05 were considered to be significantly different between insulin resistant and insulin sensitive subjects. For protein data, tests of enrichment for biological functions were performed using hypergeometric tests against biological functions from Gene Ontology Consortium (Ashburner et al., 2000), KEGG (Ashburner et al., 2000), Reactome Pathway Database (Fabregat et al., 2016), and ConsensusPathDB (Kamburov et al., 2009).

### Network-Based Integrative Analysis (iOmicsPASS)

The protein and miRNA data were merged over two biological networks including the TargetScan map ( ^2^default predictions in version 7.2) and protein–protein interaction (PPI) network, using a novel data integration strategy called iOmicsPASS (Koh et al., 2018). Briefly, the quantitative measurements of proteins and miRNAs were converted to Z-scores and they were integrated into scores of co-expression for molecular interactions (a microRNA and its target gene’s protein, or two interacting proteins) in iOmicsPASS. In the software, each interaction can be designated as positive or negative interaction by the user. Since we consider each miRNA as a translation inhibitor of its target genes in this analysis, we specified negative interactions for the TargetScan interactions. This specification instructs the software to compute the co-expression score for the interaction between miRNA and protein as the difference in Z-scores between protein and miRNA (Z-score of protein – Z-score of miRNA). For PPIs, we specified positive interactions between proteins. This leads to the co-expression score calculation as the sum of Z-scores of the two interacting proteins.

The resulting interaction-level quantitative data is used to find subnetworks of TargetScan and PPI subnetworks that are predictive of each group (OIR and LIS), using a modified version of the nearest shrunken centroid classifier, also known as Prediction Analysis of Microarray (Tibshirani et al., 2002). Final predictive subnetwork was chosen at a threshold (1.15) that resulted in the smallest average misclassification error rates over 10 sets of fivefold cross-validation (Supplementary Figure S1A).

After predictive subnetwork identification, the proteins involved in the identified predictive subnetwork were used to test enrichment of biological functions using an in-house implementation of hypergeometric tests. The part of the subnetwork associated with the identified pathways was finally visualized using Cytoscape (Shannon et al., 2003).

Once iOmicsPASS identified the protein interactome and miRNA–protein subnetwork predictive of the OIR, we incorporated the tissue of origin information for the proteins discussed above (Supplementary Figure S1B) from the Human Protein Atlas (HSPA) database. For each pair of protein and miRNA (negative co-expression) and each pair of interacting proteins (positive co-expression), we examined the tissue of origin for the proteins. A gene was considered to originate from the tissue if the tissue has evidence of mRNA and protein expression. This information enabled us to discern whether the miRNA’s translational repressive role takes place specifically in adipose tissue and liver and thus differentiate obesity-dependent and obesity-independent regulatory molecules.

## Results

### Baseline Anthropometric Data

The baseline anthropometry and fasting plasma biochemistry of study participants, consisting of eight OIR and nine LIS subjects, are shown in Table 1. One obese subject has incomplete plasma proteomic data and was excluded from the analysis. Briefly, participants in the OIR group were older, and had higher BMI, waist circumference, fasting blood glucose, insulin, triglyceride, HOMA-IR, diastolic blood pressure and alanine transaminase. As expected, OIR subjects had significantly lower HDL-cholesterol concentration than LIS subjects. Further adjustment for age did not alter the significant differences between the two groups.

**Table 1:** Baseline anthropometry and biochemistry of study participants.

	Lean insulin sensitive (*n* = 9)	Obese insulin resistant (*n* = 8)	*p*-Value
Age, years	23.2 ± 0.2	28.4 ± 1.6	0.0037
BMI, kg/m^2^	22.0 ± 0.2	29.6 ± 0.6	<0.0001
Waist circumference (cm)	79.9 ± 0.5	100.1 ± 0.8	<0.0001
Fasting glucose (mmol/l)	4.33 ± 0.06	4.71 ± 0.13	0.0149
Fasting insulin (mU/l)	4.31 ± 0.52	21.9 ± 2.4	<0.0001
HOMA-IR	0.83 ± 0.10	4.53 ± 0.41	<0.0001
Systolic blood pressure (mmHg)	110.78 ± 4.07	120.88 ± 2.69	0.0623
Diastolic blood pressure (mmHg)	60.56 ± 3.00	72.50 ± 3.41	0.0184
Fasting triglyceride (mmol/l)	0.62 ± 0.07	1.97 ± 0.27	<0.0001
HDL-cholesterol (mmol/l)	1.71 ± 0.09	1.18 ± 0.07	0.0003
Aspartate transaminase (mmol/l)	24.00 ± 3.76	37.88 ± 6.05	0.0644
Alanine transaminase (mmol/l)	18.22 ± 2.20	58.50 ± 12.61	0.0045


**Data are presented as means ± SEM; BMI body mass index; HDL high-density lipoprotein; HOMA-IR homeostasis model assessment for insulin resistance.**

### Analysis of Proteome and miRNAs

**FIGURE 1:**
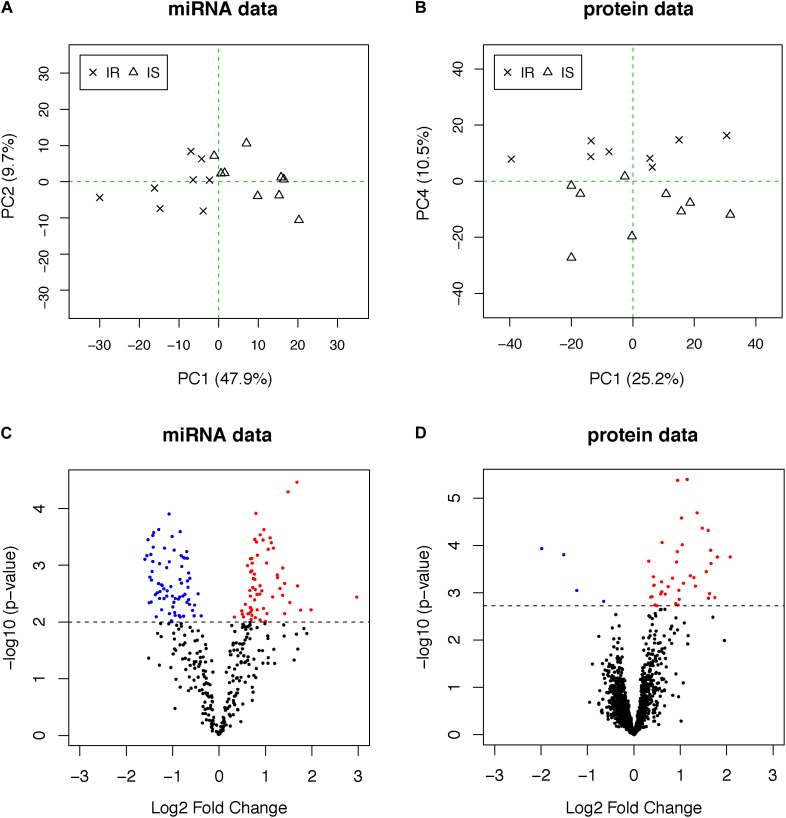
**(A,B)** PCA plots of miRNA and proteome data. OIR and LIS subjects are shown using different symbols. The vertical axis of the proteome data is the fourth principal components, indicating other axes of variation in the second and third principal components. **(C,D)** The volcano plots from the differential expression analysis. Molecules with elevated circulation levels in OIR subjects are in red, while those with reduced circulation levels in OIR subjects are in blue.

We analyzed fasting blood plasma samples using a multiplexed RT-qPCR assay platform and a liquid chromatography–mass spectrometry (LC–MS/MS) system to measure the abundance of circulating miRNAs and proteins, respectively (see section “Materials and Methods”). After data quality control and normalization, we applied PCA to each dataset to investigate whether the profiles separate the subjects into two groups in an unbiased manner. The plasma miRNAs separated the two groups more clearly (48% of total variation accounted for by PC1 in the miRNAs, Figure 1A) than the plasma proteome (10% of total variation along PC4 in the proteome, Figure 1B), suggesting that other biological factors (PC2 and PC3) influenced the inter-individual variability in the plasma protein concentrations other than obesity and IR in this data.

To identify differentially abundant proteins and miRNAs, we applied two-sample *t*-test to 368 miRNAs and 1,499 proteins. We prioritized 138 miRNAs (*p*-value < 0.01, Figure 1C) and 44 proteins (*q*-value < 0.05, Figure 1D) (Supplementary Table S1). The proportion of differentially abundant miRNAs was very high in the miRNA data, thus the *q*-value method was not able to decompose the *p*-value distribution into the null and alternative components and estimate *q*-values reliably. Therefore, we used nominal *p*-value threshold at a reasonable level, i.e., close to 50% fold change. Consistent with the PCA plots, the between-group differences in plasma miRNAs were more pronounced than those of proteins.

#### Plasma miRNAs Show a Global Shift in Circulation Levels in OIR

**FIGURE 2:**
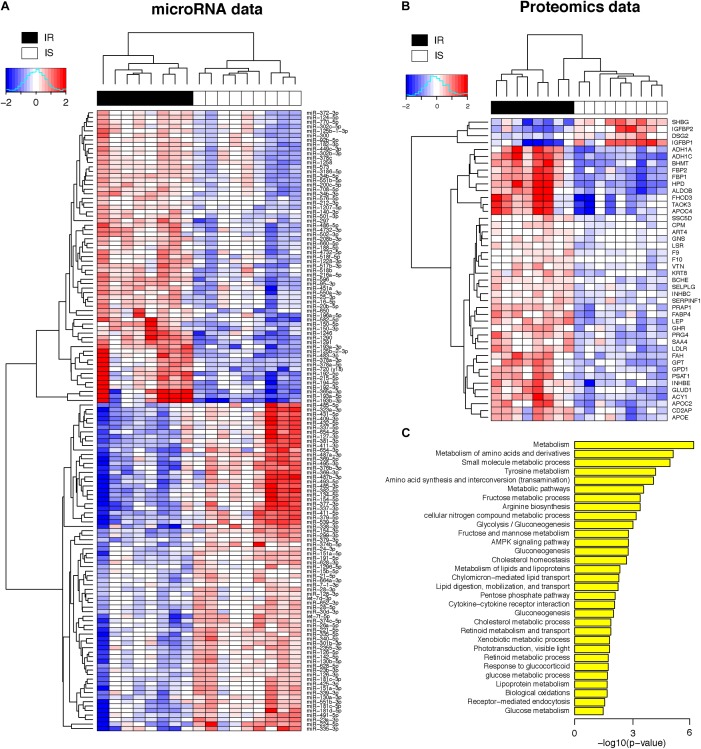
**(A)** Heatmap of plasma concentrations for the 138 miRNAs (log 2 transformed, mean-centered data). **(B)** Heatmap of plasma concentrations for the 44 proteins (same as above). **(C)** Biological processes enriched in the 44 proteins (hypergeometric *p*-value < 0.05 in the test of enrichment).

Figure 2A shows the heatmap of normalized abundance levels of the 138 miRNAs, showing significant differences between the OIR and LIS subjects. Among these, 65 species (47%) were circulating in higher levels and 73 species (53%) were circulating in lower levels in the OIR. These account for nearly a third of the miRNA species profiled on the assay, and the observation suggests that the overall balance in the miRNA secretome is perturbed in the OIR subjects compared to the LIS subjects. The notable change in miRNA secretome also indicates that miRNA-dependent intercellular signaling has changed significantly (Chen et al., 2012).

Of the 65 miRNAs in elevated circulation levels in OIR, miR-192-5p, miR-194-5p, miR-486-5p, miR-150, miR-378a-3p, miR-550a-3p, miR-16-5p, and miR-140-3p have previously been reported to be associated with IR. miR192-5p has been shown to inhibit myogenic differentiation and promote satellite cell proliferation in an animal model, and has been shown to regulate extracellular matrix components in the pathogenesis of diabetic nephropathy and non-alcoholic steatohepatitis (Liu et al., 2018). Ma et al. (2018) showed that the levels of circulating miR-150 and miR-16-3p are correlated with insulin sensitivity index measured by euglycemic clamp experiment. miR-378a is highly conserved between species, and is embedded in the first intron of the ppargc1b gene encoding PGC-1b. Both strands of miR-378a (-3p and -5p) are co-expressed with PGC-1β, for example, in the liver and during adipocyte differentiation, implicating a direct participatory role of miR-378a in the energy and fat metabolism (Krist et al., 2015). Several of these upregulated circulating miRNAs such as miR-150-5p, 16-5p, 192-5p, 451a, 486-5p, and 770-5p have been reported to be expressed in patients with T2D (He et al., 2017). Lastly, miR-193a-5p and 451a are upregulated in the skeletal muscle of T2D patients (He et al., 2017).

On the other hand, the lower circulation levels of 73 miRNAs in the OIR subjects suggest the possibility that either the biogenesis of those miRNAs is suppressed in the originating organ systems or they are secreted less as a result of IR development and reduced cell-to-cell communication in metabolically active sites, giving room for elevated protein synthesis of target genes and/or accelerated secretion into the blood.

#### Majority of Plasma Protein Candidate Markers Shows Increased Circulation Levels

Meanwhile, Figure 2B shows the normalized abundance of 40 proteins with higher circulation levels (red) and four proteins in lower circulation levels (blue) in the OIR subjects (see also Supplementary Table S1 for summary) compared to LIS subjects. The lower plasma concentrations of insulin-like growth factor binding protein 1 (IGFBP1) and sex hormone-binding globulin (SHBG) in the OIR subjects are consistent with the literature (Rajwani et al., 2012; Wallace et al., 2013). IGFBP2 is secreted by differentiating white preadipocytes, and *in vitro* data suggest that IGFBP2 directly prevents adipogenesis and improves insulin sensitivity (Wheatcroft et al., 2007).

The significance of lower concentration of plasma desmoglein-2 (Dsg2) among the OIR subjects compared to the LIS subjects has not been discussed extensively in the literature. Dsg2 is highly expressed in epithelial cells, and together with other members of the Dsg family, they are involved in the regulation of cell cohesion in keratinocytes. Dsg2 is the only desmoglein expressed in the heart, has been shown to play an important role in cardiomyocyte cohesion and function (Schlipp et al., 2014). It has been postulated that obesity leads to lipotoxic cardiomyopathy, interstitial fibrosis, and inflammation leading to dysfunctional desmosomal proteins such as Dsg2 (Samanta et al., 2016). More recently, Dsg2 has been shown to regulate intestinal barrier function beside its adhesion function and its dysfunction might underlie the inflammatory bowel conditions (Ungewiss et al., 2017).

**FIGURE 3:**
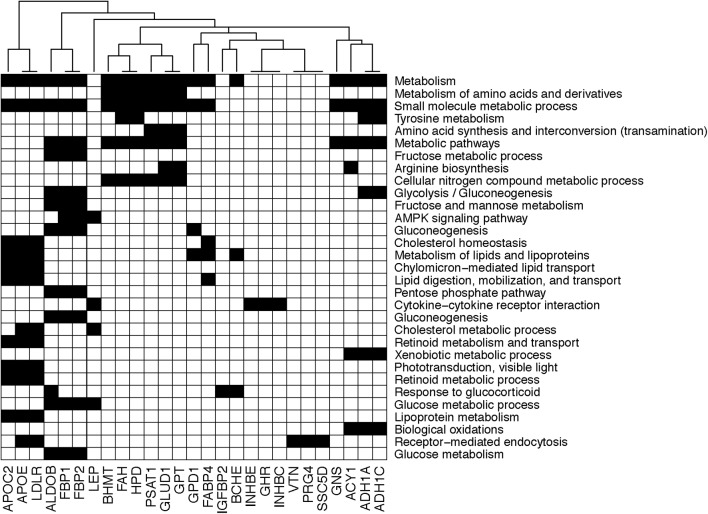
Diagram showing the membership of individual genes in biological processes. Black square implies that the corresponding gene in the column is involved in the biological process in the row.

On the other hand, the plasma concentration of remaining 40 plasma proteins were higher in the OIR than LIS subjects. The dominant proportion of the miRNA species with lower circulation levels in OIR also indicates that the lower circulatory miRNA signatures are more related to the protein secretome changes in the OIR subjects. The proteins with higher concentration in the OIR subjects are involved in a variety of metabolic and endocytic functions (see Figure 2C and Supplementary Table S2). These metabolic functions include amino acid metabolism (BHMT, FAH, GLUD1, GPT, HPD, PSAT1), glucose metabolism (ALDOB, FBP1, FBP2), lipid transport and cholesterol metabolism (APOC2, APOE, LDLR), lipid and lipoprotein metabolism (GPD1, FABP4), and other cellular functions such as biological oxidation (ACY1, ADH1A, ADH1C), cytokine receptor interaction (GHR, INHBC, INHBE), and receptor-mediated endocytosis (PRG4, SSC5D, VTN) (see Figure 3).

In addition, plasma concentrations of leptin (LEP) and proline-rich acidic protein 1 (PRAP1) are known to be elevated in obese subjects (Jequier, 2002). Plasma leptin, secreted by adipose tissue, is in higher concentrations among the OIR subjects with a high degree of adiposity in our data, indicating leptin resistance. Circulating PRAP1, secreted by liver, gastrointestinal tract and kidney, has negative correlation with insulin sensitivity and its concentrations decreased with weight loss intervention among obese individuals (Oller Moreno et al., 2018). Taken together, our proteomic profiling captures a comprehensive set of key plasma proteome associated with IR and obesity together.

#### Tissue of Origin Analysis Reveals Obesity-Dependent and Independent Protein Signatures

We also examined the potential tissue(s) of origin for each prioritized protein by querying the RNA-level and protein-level expression of the 44 proteins in the HSPA database. If a tissue is the major site of synthesis and secretion for a protein, evidenced by the mRNA and protein expression in the tissue, then we can make an educated guess for the tissue(s) of origin for the regulatory action by miRNA on their target genes. Thus, the tissue of origin information will be useful in determining which of the 44 proteins are secreted primarily by adipose tissues, i.e., in obesity-dependent manner. Likewise, the information will also discern which proteins are secreted by other metabolic organ systems such as liver and skeletal muscle, i.e., in obesity-independent manner.

Supplementary Table S3 shows that some proteins are secreted from adipose tissues: apolipoprotein E (APOE), carboxypeptidase M (CPM), fatty acid binding protein 4 (FABP4), fumarylactoacetate hydrolase (FAH), growth hormone receptor (GHR), glucosamine (*N*-acetyl)-6-sulfatase (GNS), glycero-3-phosphate dehydrogenase (GPD1), glutamic-pyruvic transaminase (GPT), low density lipoprotein receptor (LDLR), leptin (LEP), and serpin family F member 1 (SERPINF1). However, it is also possible that some of those proteins may be secreted from other endocrine tissues, liver, gastrointestinal tract, and kidney.

We next focused on the proteins synthesized in the liver, as liver is a major contributor to the human secretome of metabolic enzymes. If found to be exclusively secreted from liver, this will indicate that the proteins represent the secretome shift that is likely due to fat infiltration of the liver and subsequent IR in liver. Among the four proteins with lower circulation levels in OIR, sex hormone-binding globulin (SHBG) and insulin-like growth factor binding proteins 1 (IGFBP1) are almost exclusively synthesized in liver, and IGFBP2 protein are known to be produced by a multitude of organs including liver, pancreas, and immune cells based on the HSPA.

Compared to the LIS subjects, OIR subjects have higher plasma levels of alcohol dehydrogenase 1A (ADH1), aldolase (ALDOB, or fructose bisphosphatase B), apolipoprotein C2, apolipoprotein C4, apolipoprotein E (APOE), ADP-ribosyltransferase 4 (ART4), butyrylcholinesterase (BCHE), betaine-homocysteine *S*-methyltransferase (BHMT), fructose-1,6-bisphosphatase 1 (FBP1), coagulation factors IX and X, 4-hydroxyphenylpyruvate dioxygenase (HPD), inhibin-beta C and E chain (INHBE, INHBC), proteoglycan 4 (PRG4), serum amyloid A-4 protein precursor (SAA4), and vitronectin (VTN). These plasma proteins are involved in the majority of biological functions mentioned above, including glycolysis, glucose and protein metabolism, lipid transport, and other responses to inflammation and endocytic functions (Figure 3). The overall plasma protein landscape matches that of the literature, which has indicated that IR is associated with up-regulation of glycolytic enzymes, aberration in coagulation pathway and pro-inflammatory immune response in circulating blood (Carayol et al., 2017; Sun et al., 2018).

Interestingly, fructose-2,6,bisphosphatase 2 (FBP2, also known as FBPase-2) was the only protein with increased levels in circulation among the OIR subjects that uniquely originate from skeletal muscles, a major site of glucose uptake and metabolism. The cellular concentration of FBP2 is regulated by the enzyme 6-phosphofructo-2-kinase/fructose-2,6-bisphosphatase (PFK2/FBP2), and is critical in determining the activity of 6-Phosphofructo-1-kinase (PFK1) in the regulation of glycolysis and gluconeogenesis (Zhao et al., 2012).

### Network-Based Predictive Analysis Using Integrated miRNA and Proteome Data

#### Analysis Strategy for Hypothesis-Driven Data Integration

The plasma proteins and miRNAs discussed above originate from various organ systems, and thus the relationship between the two secretomes in the circulation remains elusive. In particular, the origins of secreted miRNAs are largely unknown, and thus the integrative analysis of the two data sets incorporating the tissue of origin information for proteins can provide possible cues for the primary sites of secretion for the miRNAs. Our basic principle of integration is the following: each miRNA contributes to the repression of synthesis of the proteins of its target genes in the originating organ(s), and the circulation levels of the two molecules are negatively correlated in each individual subject.

To this end, we analyzed the miRNA and proteomic data using iOmicsPASS, a novel network-based predictive analysis tool (Koh et al., 2018), and combined the results with tissue of origin information for the proteins. The iOmicsPASS converts multi-omic measurements into co-expression scores of interacting molecules on a biological network of choice, and finds predictive subnetworks for phenotypic groups (e.g., OIR and LIS). The original implementation of iOmicsPASS integrates DNA copy number, mRNA transcript abundance, and protein abundance, and thus it only searches for positive co-expression patterns, where the abundance of two interacting molecules are high or low together in the same direction. For the integration of miRNAs and proteins, we assume that a high level of a circulating miRNA implies increased biogenesis of the miRNA in the originating organ system, which in turn actively represses protein translation of its target mRNA, resulting in a low level of protein synthesis and secretion. Therefore, we revised our algorithm to account for *negative co-expression* between miRNAs and proteins, while still searching for positive co-expression of physically interacting proteins.

#### Network-Based Integrative Analysis Identifies Potential Origin of Secreted miRNAs and Sites of Regulatory Action

Supplementary Figure S1A shows the misclassification errors based on cross-validation in the selection of the optimal size of a predictive subnetwork. Since the number of samples is relatively small, we repeated this exercise 10 times and chose the threshold that gave the lowest average misclassification error rates, resulting in 98 relationships (85 miRNA–protein regulation, 13 PPIs). The cross-validation error plots consistently showed low-test errors at the optimal point and the probability of classification was highly consistent with their actual groups within the training data, suggesting that there is a robust co-expression subnetwork signature, predictive of OIR in this data. The optimized co-expression profiles clearly distinguished the OIR from LIS subjects.

**FIGURE 4:**
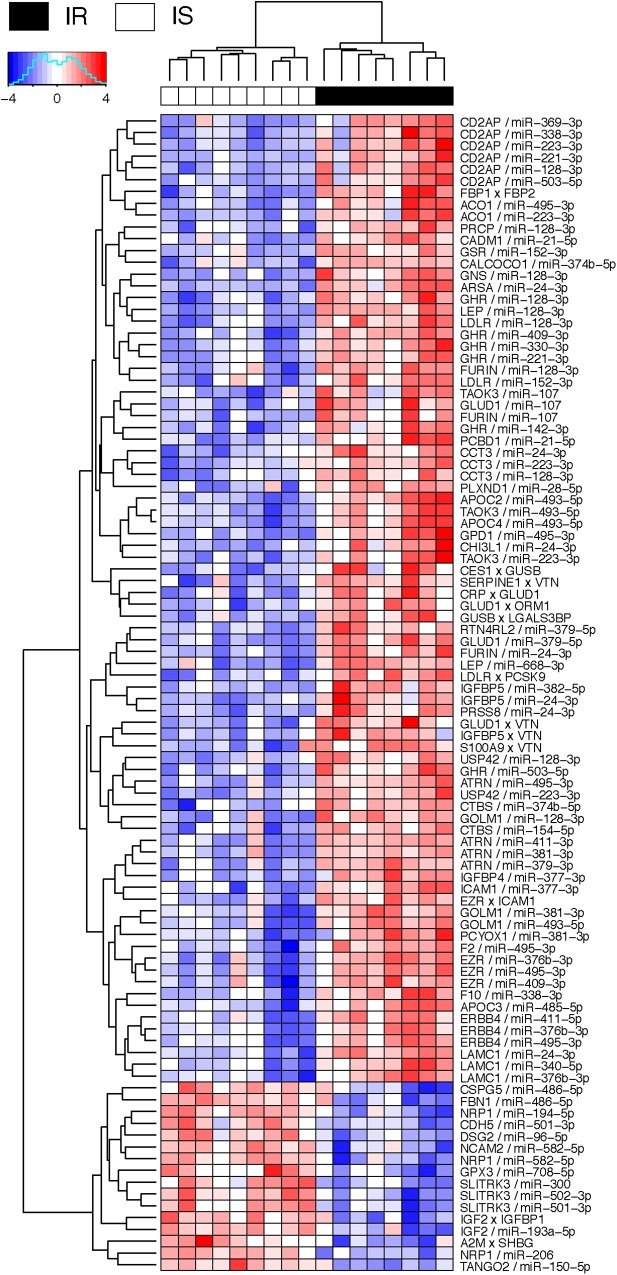
Heatmap of interaction score for miRNA–protein and protein–protein pairs. Only the pairs that are predictive of OIR and LIS phenotype are shown. For miRNA–protein pairs in a subject, red color indicates that the protein level is greater and miRNA is lower in the sample than the average levels across the subjects. For protein–protein pairs, red color means the circulation levels are higher for both proteins in the sample than the average levels.

**FIGURE 5:**
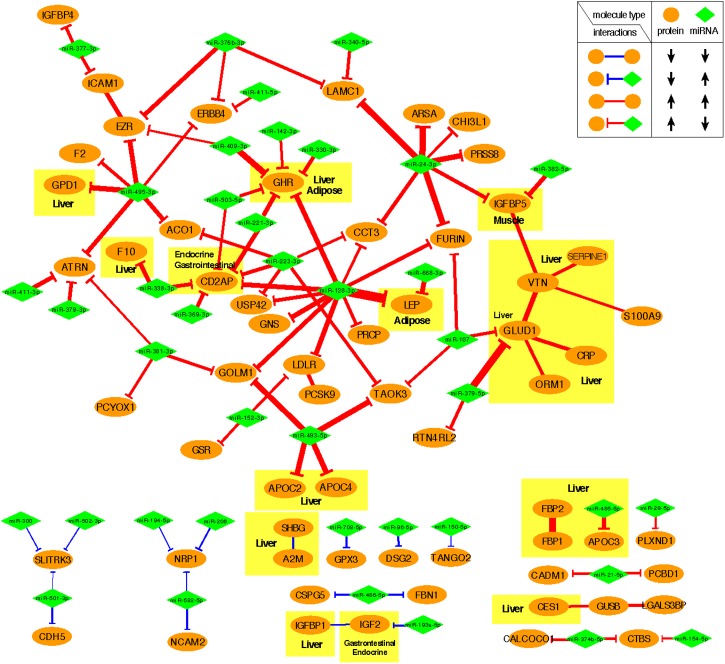
Predictive subnetwork surrounding the proteins with tissue of origin information highlighted in yellow boxes. Proteins and miRNAs are shown in ellipsoids and diamonds, respectively. The proteins that are ubiquitously expressed across multiple organ systems were not highlighted.

Figure 4 shows the negative co-expression profiles of miRNA–protein pairs and positive co-expression profiles of interacting proteins, both of which are predictive of OIR status. Simultaneously, Figure 5 presents a graphical visualization of the selected subnetwork (Supplementary Table S4 for the details of the selected network data). We observed 71 negative co-expression pairs of miRNA with proteins, with decreased miRNA secretion and increased protein secretion levels in the OIR subjects. TargetScan predicted several notable miRNAs as regulators of a multitude of protein markers discussed above. miR-128-3p, which was secreted less in IR, is predicted to regulate synthesis of LEP and GHR in adipose tissues, CD2AP in endocrine and gastrointestinal tissues, and LDLR in a large variety of organs. Sud et al. (2017) previously showed that mice fed high-fructose diet for 4 weeks showed downregulation of liver miR-128-3p level, and the computing prediction further showed that the miR-128-3p targets gene of IRS-1, FOXO1 and MTTP. miR-338 also regulates the synthesis of coagulation factor F10 in liver and CD2AP. miR-493-5p targets APOC2 and APOC4, uniquely produced in liver, and miR-379-5p regulates the production of GLUD1 in liver which is an important enzyme in the urea synthesis. In contrast, de Guia et al. (2015) demonstrated that hepatic miR-379 expression in was lower in lean (mean BMI 23.8 ± 0.8) compared to obese (mean BMI 45.2 ± 6.5) individuals, and that mir-379 plays a key role in the pathogenesis of the glucocorticoid-dependent hypertriglyceridemia.

miR-24-3p, miR-223-3p, and miR-495-3p also had multiple connections to various plasma proteins, but most of the target proteins were ubiquitously expressed across organ systems (RNA-level) and therefore it was difficult to designate potential sites of regulatory action for those miRNAs. However, there were some other interesting connections. TargetScan predicted miR-24-3p to regulate IGFBP5, uniquely produced in muscle tissues in males, and the data suggests that the increased secretion level of IGFBP5 in IR is partly attributable to the reduced secretion levels of miR-24-3p. Likewise, miR-495-3p is predicted to regulate glycerol-3-phosphate dehydrogenase 1 (GPD1), produced in liver, and the increased secretion of the latter in IR can be partially explained by the decreased secretion level of the former.

#### Integrative Analysis Also Identifies the Protein Interactome Predictive of IR

In the network-based predictive analysis, we also observed 13 pairs of proteins that are known to bind to one another in the intracellular space. The most notable predictive sub-interactome was anchored around glutamate dehydrogenase 1 (GLUD1) and vitronectin (VTN), both produced mainly in liver. A hemopexin family glycoprotein VTN is most famously known to bind to plasminogen activator inhibitor-1 (PAI-1 or SERPINE1) to stabilize the active conformation of PAI-1 in modulating fibrinolysis and cell migration (Zhou et al., 2003), which is a part of the selected interactome. The higher levels of VTN and PAI-1 in the OIR subjects also indicates the higher risk of cardiovascular thromboembolic events among people with obesity and insulin resistance. GLUD1 also binds to orosomucoid 1 (ORM1) and C-reactive protein (CPR), indicating that elevated secretion of these proteins signals the activation of acute-phase proteins and pro-inflammatory state of the OIR subjects.

Another interesting predictive protein interactome is that of fructose 1,6-bisphosphatases (FBP1) and a bifunctional enzyme fructose 2,6-bisphosphatase isozyme 2 (FBP2), both with higher secretion levels in OIR. Synthesized and secreted from liver, the two proteins are expressed in all metabolic organ tissues (Supplementary Table S3), including pancreas (Supplementary Figure S1), indicating their elevated binding activity and regulation of glycolysis and gluconeogenesis in those sites.

On the other hand, IGFBP1-IGF2 and A2M-SHBG are two pairs of physical binding partners that are simultaneously downregulated in OIR, and both are likely liver-specific interactions based on the origin of IGFBP1 and SHBG. However, the magnitude of co-expression difference between OIR and LIS was of a minor magnitude (see IR centroid column of Supplementary Table S4), and therefore we did not pursue further interpretation on these interactions.

## Conclusion

The plasma secretome captures a complex mixture of biomolecules secreted from various organ systems into circulation. Despite having a small sample size, our data reflect this phenomenon by showing protein biomarkers suspected to originate from a multitude of metabolic organ systems, immune cells, and other sites such as brain and heart. Whether the elevated secretion levels of proteins in the OIR subjects are directly influenced by the overall balance shift of miRNAs at the site of protein synthesis remains to be thoroughly validated in model systems and through replication in independent studies of a sufficiently large sample size. However, as far as we know, our work is one of the first studies that profiled plasma secretome using both -omics platforms on the human plasma samples from OIR and LIS subjects. Accordingly, we explored the connections between the two types of secretomes via a novel predictive analysis framework iOmicsPASS and tissue of origin and expression information from independent source (HSPA). This systems-level analysis allowed us to make an educated guess on a few cases of miRNA-mediated translation regulation of plasma protein levels and protein interactions in OIR in specific tissues.

Two recent studies have attempted to integrate plasma proteome and genomic data, where the protein expression was measured using a multiplexed aptamer-based SomaScan proteomic assay. Sun et al. (2018) identified 1927 genetic associations with 1478 plasma proteins from 3,301 healthy participants from the INTERVAL study. Similar to our findings, they also reported that the plasma concentration of IGFBP1, IGFBP2, SHBG and desmoglein-2 are negatively correlated with BMI. Carayol et al. (2017) provided an integrative analysis of changes in the plasma proteomes with gene expression from 494 obese subjects before and after a weight loss intervention. At baseline, the authors found that the four plasma proteins of IGFBP1, IGFBP2, SHBG, and desmoglein-2 are also negatively correlated with BMI. These results provide us with confidence that the four mentioned plasma proteins are unequivocally altered (reduced plasma concentration) in obese subjects with insulin resistance.

A lingering question we did not address in our analysis is the connection between the miRNA species that were secreted more in the OIR subjects and the proteome changes. It is plausible that exosome and RNA binding protein-based export of a specific set of miRNA species have increased in certain tissues to support other biological functions to address the shortage of glucose and meet the metabolic needs for energy expenditure. However, this complex mechanism is only sparsely mapped in the current literature, hence we leave this for future work.

Our work illustrates the opportunities and challenges of integrating two different -omics scale data too, especially in the context of analyzing the secretome. In our case, our data analysis framework using protein interactome information was able to identify two small protein interactomes in the liver, one implicated in elevated inflammatory state and the other directly involved in glycolysis and gluconeogenesis. TargetScan-based connections also highlighted miRNA species with lower secretion levels associated with elevated secretion levels of the target proteins. However, it was also important to recognize that these interactions likely occur in the intracellular environment prior to being secreted into the blood. Therefore, the tissue of origin information from the HSPA was crucial for proper prioritization of the co-expression patterns observed in the secretome, especially allowing us to distinguish the miRNA species regulating protein synthesis in adipose tissues versus other metabolic organs such as liver. As such, we recommend that this site of origin aspect be taken into account for data interpretation in future clinical studies.

From a therapeutic standpoint, our integrative analysis clearly demonstrated that IR is a manifestation of mature systemic metabolic dysfunctions rather than an isolated event of disruption in cellular glucose uptake. Therefore, IR and subsequent dysregulation in cellular glucose uptake is accompanied by perturbations in an array of other biological functions in the system. As such, the search for future therapeutic options or the therapeutic strategy may have to focus on jointly modulating other biological functions to promote the balance in the metabolism and transport of different fuel sources, while improving insulin sensitivity and restoring glucose uptake. To this end, we expect systems biology-based approaches hold great potential to identify related and actionable targets for successful treatment of IR in the future.

## Ethics Statement

This study was carried out in accordance with the recommendations of ‘name of guidelines, name of committee’ with written informed consent from all subjects. All subjects gave written informed consent in accordance with the Declaration of Helsinki. The protocol was approved by the National Healthcare Group Domain Specific Review Board (Singapore).

## Author Contributions

CK and ST conceived, designed, and supervised the project. TL and EPR executed the project. CK supervised the plasma proteomics. HyC and HK performed bioinformatics analysis of microRNA and proteomics data, and multi-omics data integration analysis. HyC and CK wrote the manuscript. HyC, CK, BH, and GR reviewed the final manuscript. All authors have read and approved the manuscript.

## Conflict of Interest Statement

GR and BH are full-time employees of Janssen Research & Development, United States. LZ and HyC are employees of MIRXES, Pte. Ltd., Singapore. The remaining authors declare that the research was conducted in the absence of any commercial or financial relationships that could be construed as a potential conflict of interest.

## Footnotes

1 http://cran.r-project.org

2 www.targetscan.org

## Supplementary Material

Figure S1 Integrative analysis with iOmicsPASS. **(A)** IR prediction accuracy evaluation in iOmicsPASS in terms of misclassification errors (test errors) through 10 rounds of fivefold cross-validation. Barplot shows the probability of IR for the 17 subjects using the best predictive model (training data). **(B)** Workflow of iOmicsPASS with incorporation of tissue-of-origin analysis. A miRNA–protein regulation was considered to originate from an organ if the protein was annotated to be synthesized from the organ according to the HSPA database. A protein–protein interaction (PPI) was considered to occur in an organ if both proteins were annotated to be synthesized from the organ.

Table S1 Normalized microRNA and proteomics data and results of differential expression analysis.

Table S2 Biological functions enriched in the plasma proteins differentially abundant in the OIR subjects compared to the LIS subjects.

Table S3 Tissue of origin analysis for differentially abundant plasma proteins based on RNA and protein-level evidence in the Human Protein Atlas database.

Table S4 List of protein-protein interactions (positive interaction) and miRNA-protein regulatory relationships (negative interaction) predictive of OIR and LIS.

## Abbreviations

BMI: body mass index
HDL: high density lipoprotein
HOMA: homeostatic model assessment
IR: insulin resistance
LIS: lean insulin sensitive
OIR: obese insulin-resistant
T2D: type 2 diabetes
