# Supplementary figures and images for: Plasma Protein and MicroRNA Biomarkers of Insulin Resistance: A Network-Based Integrative -Omics Analysis
Source: Front Physiol. 2019 Apr 5;10:379. doi: 10.3389/fphys.2019.00379 (PMC6460474; doi:10.3389/fphys.2019.00379)

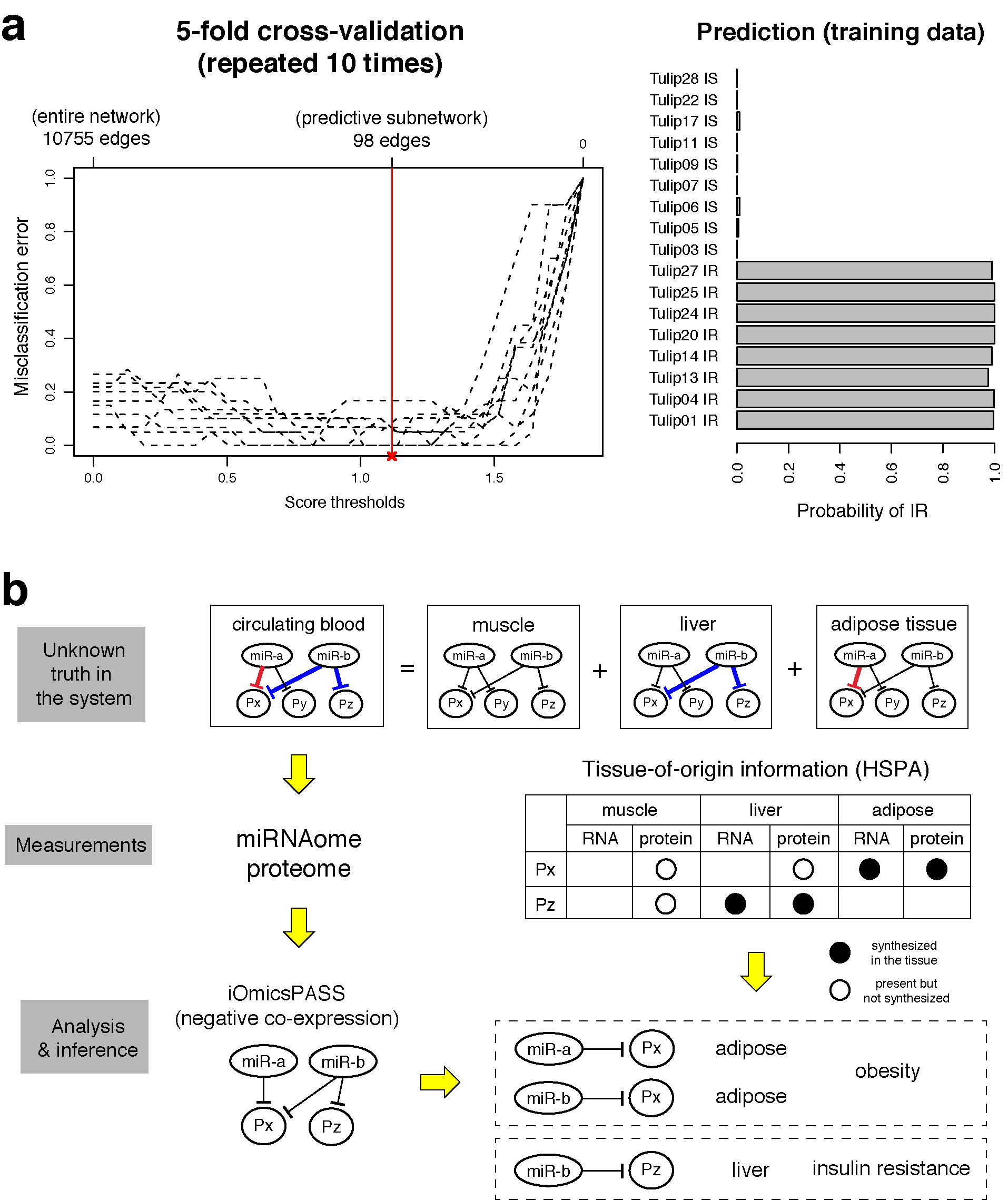

Supplement: Figure S1 — Integrative analysis with iOmicsPASS. (A) IR prediction accuracy evaluation in iOmicsPASS in terms of misclassification errors (test errors) through 10 rounds of fivefold cross-validation. Barplot shows the probability of IR for the 17 subjects using the best predictive model (training data). (B) Workflow of iOmicsPASS with incorporation of tissue-of-origin analysis. A miRNA–protein regulation was considered to originate from an organ if the protein was annotated to be synthesized from the organ according to the HSPA database. A protein–protein interaction (PPI) was considered to occur in an organ if both proteins were annotated to be synthesized from the organ. [file Image_1.tif]
